# Artificial Venus Flytraps: A Research Review and Outlook on Their Importance for Novel Bioinspired Materials Systems

**DOI:** 10.3389/frobt.2020.00075

**Published:** 2020-07-08

**Authors:** Falk J. Esser, Philipp Auth, Thomas Speck

**Affiliations:** ^1^Plant Biomechanics Group and Botanic Garden, University of Freiburg, Freiburg, Germany; ^2^Cluster of Excellence livMatS @FIT, Freiburg Center for Interactive Materials and Bioinspired Technologies, University of Freiburg, Freiburg, Germany; ^3^Freiburg Center for Interactive Materials and Bioinspired Technologies (FIT), Freiburg, Germany; ^4^FMF, Freiburg Materials Research Center, University of Freiburg, Freiburg, Germany

**Keywords:** artificial Venus flytrap, artificial, materials systems, biomimetics, demonstrators, embodied intelligence

## Abstract

Bioinspired and biomimetic soft machines rely on functions and working principles that have been abstracted from biology but that have evolved over 3.5 billion years. So far, few examples from the huge pool of natural models have been examined and transferred to technical applications. Like living organisms, subsequent generations of soft machines will autonomously respond, sense, and adapt to the environment. Plants as concept generators remain relatively unexplored in biomimetic approaches to robotics and related technologies, despite being able to grow, and continuously adapt in response to environmental stimuli. In this research review, we highlight recent developments in plant-inspired soft machine systems based on movement principles. We focus on inspirations taken from fast active movements in the carnivorous Venus flytrap (*Dionaea muscipula*) and compare current developments in artificial Venus flytraps with their biological role model. The advantages and disadvantages of current systems are also analyzed and discussed, and a new state-of-the-art autonomous system is derived. Incorporation of the basic structural and functional principles of the Venus flytrap into novel autonomous applications in the field of robotics not only will inspire further plant-inspired biomimetic developments but might also advance contemporary plant-inspired robots, leading to fully autonomous systems utilizing bioinspired working concepts.

## Introduction

In the last decade, the topics of soft robotics and soft machines have tremendously grown as research fields. The field of compliant robots has grown tremendously from the early beginnings of compliant-like actuation for bioinspired robots fitted with McKibben muscles in the early 1950's and 1960's (Agerholm and Lord, 1961; Schulte Jr H. F., 1961). The hard but inflatable McKibben muscles paved the way for inflatable and flexible micro-actuators (Baldur and Blach, 1985; Suzumori et al., 1991), which made compliant actuators considerably smaller, inspiring the development of flexible continuum robots, with Robinson and Davies (1999) highlighting the state of the art, and further to flexible silicone-based robots such as the iconic multigait soft robot of Shepherd et al. (2011). Bridging the gap from compliant to fully flexible autonomous soft machines, systems were developed such as Kim's autonomous meshworm (Seok et al., 2010; Kim et al., 2013) or the now iconic entirely soft, autonomous robot “octobot” by Wehner et al. (2016). Spanning decades, the research achieved a transition from hard robots with soft actuation to entirely soft systems. These systems were made possible by utilizing various smart and partially soft materials as actuators, such as liquid crystal elastomers (LCEs) (Wani et al., 2017), shape memory alloys (SMAs) (Kim et al., 2013), and polymers (Mather et al., 2009; Behl et al., 2013; Meng and Li, 2013; Besse et al., 2017), electroactive polymers (e.g., DEA, Pelrine et al., 2000; Wang et al., 2019, and IPMC, Shahinpoor, 2011), and materials with thermal (Behl et al., 2013), and humidity responsiveness such as hydrogel (Athas et al., 2016). The newest systems are capable not only of soft actuation but also of “soft sensing” by utilizing soft materials such as conductive elastomers or silicones and/or soft and flexible channels filled with liquid metals (e.g., EGaIn, consisting of a mixture of gallium, indium, and tin) forming soft sensors (Kumar et al., 2019). Materials systems are also available, functioning as stretchable electroluminescent skin; these are able to emit light actively, sense deformation, and withstand surface expansion of over 600% (Larson et al., 2016; Zhou et al., 2019). Such extraordinary developments enable a new age of sensing and environment-adaptive robots.

A novel field of soft robotics and soft machines has also emerged within the last few years, namely, that of plant-inspired robotics, focusing on the implementation of the functional principles of plants. These systems utilize structural and functional principles of plants to move, harvest energy, and sense the environment. Plants in particular are well-suited as models for adaptable materials systems that consist of hierarchically structured materials systems with various functions that span several orders of magnitude and that show adaptations to changing environmental conditions, for example, through growth processes and material restructuring.

Since plants are sedentary photoautotrophic organisms with the ability to self-reproduce organic molecules (through photosynthesis), locomotion is not strictly necessary. If environmental conditions change, they adapt by changing their physiology and behavior in order to improve their interception of solar radiation and their uptake of ions from the air and the soil, respectively. By means of the exploration and colonization of habitats, plants are able to overcome obstacles, penetrate into hard media, and even move within it, for example, roots within soil (Roy and Bassham, 2014; Sadeghi et al., 2014, 2017). Sensing, selection processes, and reactions to changing conditions are accomplished in plants without a central control unit (i.e., a brain). This raises the possibilities of using plants as role models for autonomous robots whereby the complexity of the overall system can be reduced by eliminating the need for a central control unit and replacing it with a distributed, plant-like, cue-sensitive system that reacts only to certain stimuli.

Currently, soft robotic systems are available based on plant organs such as tendrils, roots, and leaves (Laschi et al., 2017; Wang et al., 2018; Must et al., 2019; Mazzolai et al., 2020). The leaf-inspired systems are particularly interesting not only as role models for energy harvesters (Liu et al., 2016; Jie et al., 2018; Meder et al., 2018, 2020) but also for fast motions in examples of soft machines inspired by carnivorous plants (Esser et al., 2019). The carnivorous plants *Dionaea muscipula* (Venus flytrap) and *Aldrovanda vesiculosa* (waterwheel plant) have inspired a number of biomimetic robots and facade shading systems for elastic architecture during the last decade (Schleicher et al., 2015; Körner et al., 2018; Knippers et al., 2019). Darwin (1875) was fascinated by *D. muscipula* and called it “one of the most wonderful plants in the world.” Therefore, it is not surprising that many attempts have been made to create an artificial trap inspired by the movements of the *D. muscipula*.

**Figure 1:**
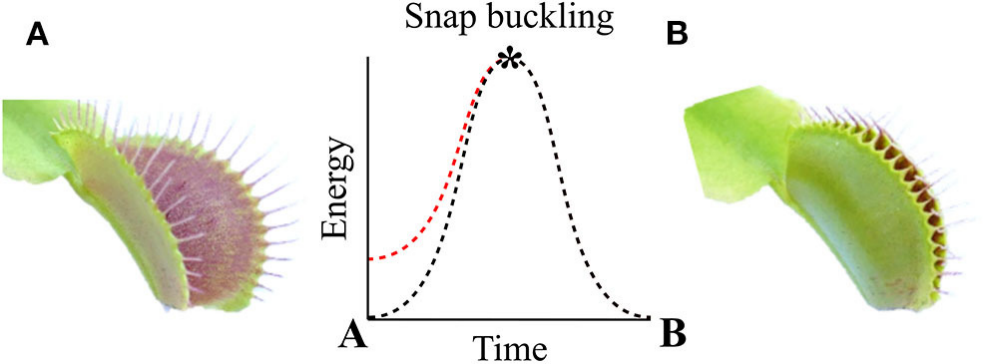
Two low-energy states of *Dionaea muscipula* before **(A)** and after **(B)** snap buckling. The trap lobes show a concave spatial curvature in the open state **(A)**, which undergoes rapid curvature inversion during snap buckling when closing to a convex curvature. Original figure based on concepts presented of Westermeier et al. (2018).

The natural habitats of the biological role model for these systems, namely, *D. muscipula*, are nutrient-poor environments such as bogs. To meet its nutrient demands, it catches small arthropods and digests them within its traps. One plant can grow up to 10 leaves with traps that are ~20 mm long, each consisting of two lobes. The lobes are connected via a midrib, with three to four trigger hairs being present on the inside of each lobe. Trap closure is triggered when prey enters the trap and stimulates at least one of the trigger hairs inside the trap twice within a certain time frame (20–30 s at room temperature, Hodick and Sievers, 1989). Water displacement followed by the release of stored elastic energy takes place, leading to the closing movement of the trap leaves within 0.1–0.5 s (Forterre et al., 2005; Poppinga et al., 2018). Trap lobes that are open and ready to snap have a typical concave spatial curvature (as seen from the outside) and undergo rapid curvature inversion releasing the stored energy (snap buckling) when closing. Therefore, the leaves can be described as bistable systems with two low-energy states (Figure 1) (Poppinga and Joyeux, 2011; Westermeier et al., 2018; Sachse et al., under revision). The energy consumption of *D. muscipula* for one trap closure is ~300 μmol ATP, equivalent to 9.66 J (Jaffe, 1973) (ATP hydrolysis consumes roughly 30.5 kJ/mol (Rosing and Slater, 1972). Reopening, after prey capture and digestion, occurs over 1–2 days Fagerberg and Howe, 1996; Volkov et al., 2014; Poppinga et al., 2016, 2018). It is controlled either by irreversible growth processes (Ashida, 1934) or by hydrostatic pressure changes within the lobes (Markin et al., 2008). In comparison, the trap closure of its carnivorous sister species, the waterwheel plant *A. vesiculosa*, utilizes active hydraulics, elastic relaxation, and kinematic amplification via midrib bending deformation (Westermeier et al., 2018), whereas the Venus flytrap employs an initial hydraulic deformation, followed by elastic instability (Sachse et al., under revision). The various mechanical principles for snapping are related to physical limits such as trap size and tissue thickness, which both fundamentally differ in the two traps types (Westermeier et al., 2018, 2019). The kinematic coupling of the midrib bending and trap closure has inspired the development of the Flectofold facade shading system, which incorporates a bioinspired kinetic curved-line folding system with distinct flexible hinge zones actuated with pneumatic cushion bending the midrib (Körner et al., 2018; Saffarian et al., 2019).

**Figure 2:**
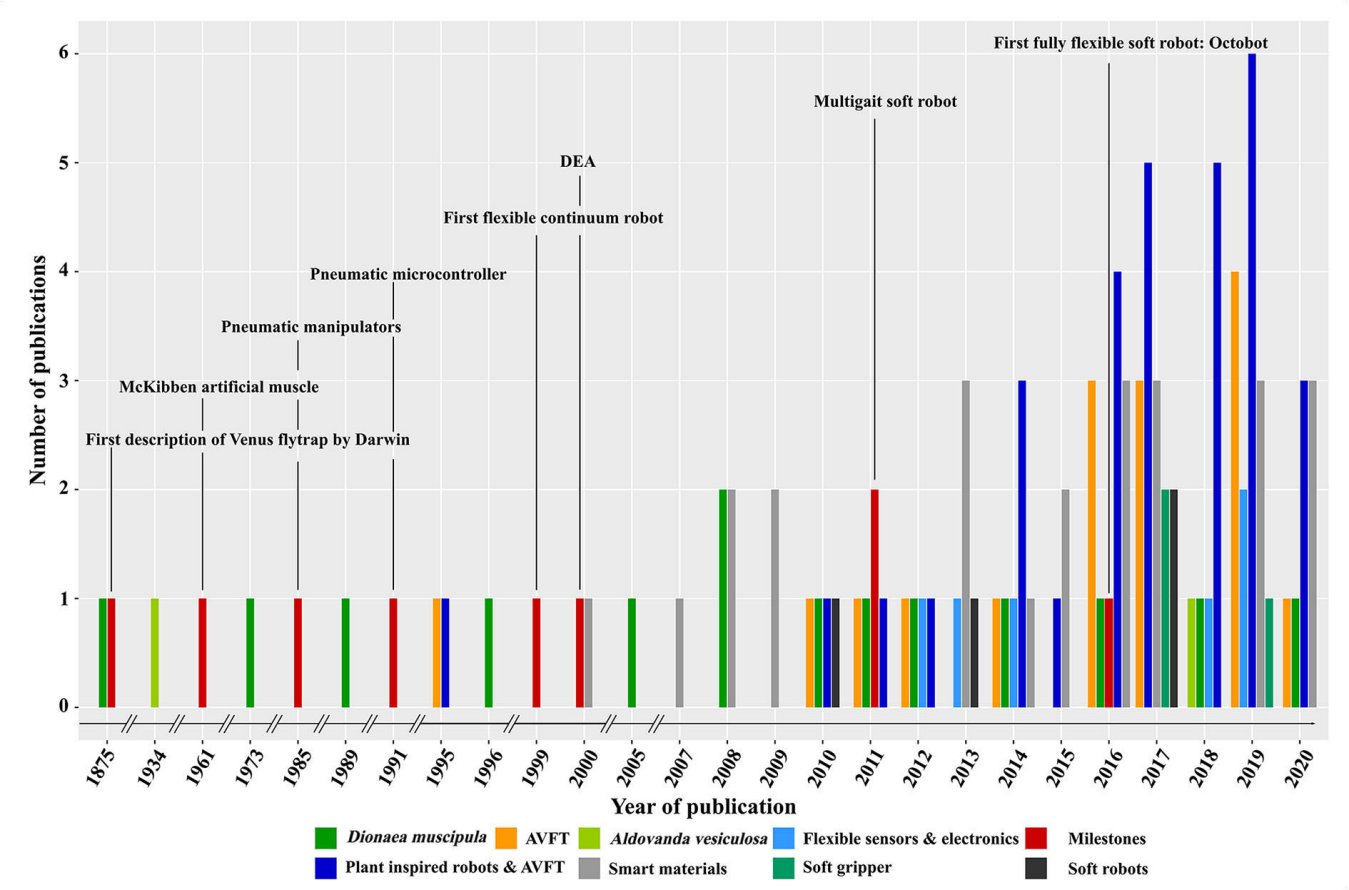
Bibliographic overview of cited publications, highlighting the number of cited publications concerning research about Venus flytraps, *Aldrovanda vesiculosa*, smart materials (*inter alia*: unit cells, logical metamaterials, and self-healing materials), flexible sensors and electronics, soft robots, and plant-inspired robotics including AVFT over the last 145 years since the first description of the Venus flytrap by Darwin in 1875. The numbers are set into relation to noteworthy milestones within these fields. AVFTs were developed within the last 25 years. Shahinpoor and Thompson (1995) were the first to consider theoretically developing an AVFT based on IPMC, and in 2011, Shahinpoor published a paper on an actual IPMC-based AVFT. Within the last decade, publication numbers have risen from one in 2010 to five in 2019, highlighting the growing interest in AVFT systems as platforms or showcases for novel materials developments.

**Figure 3:**
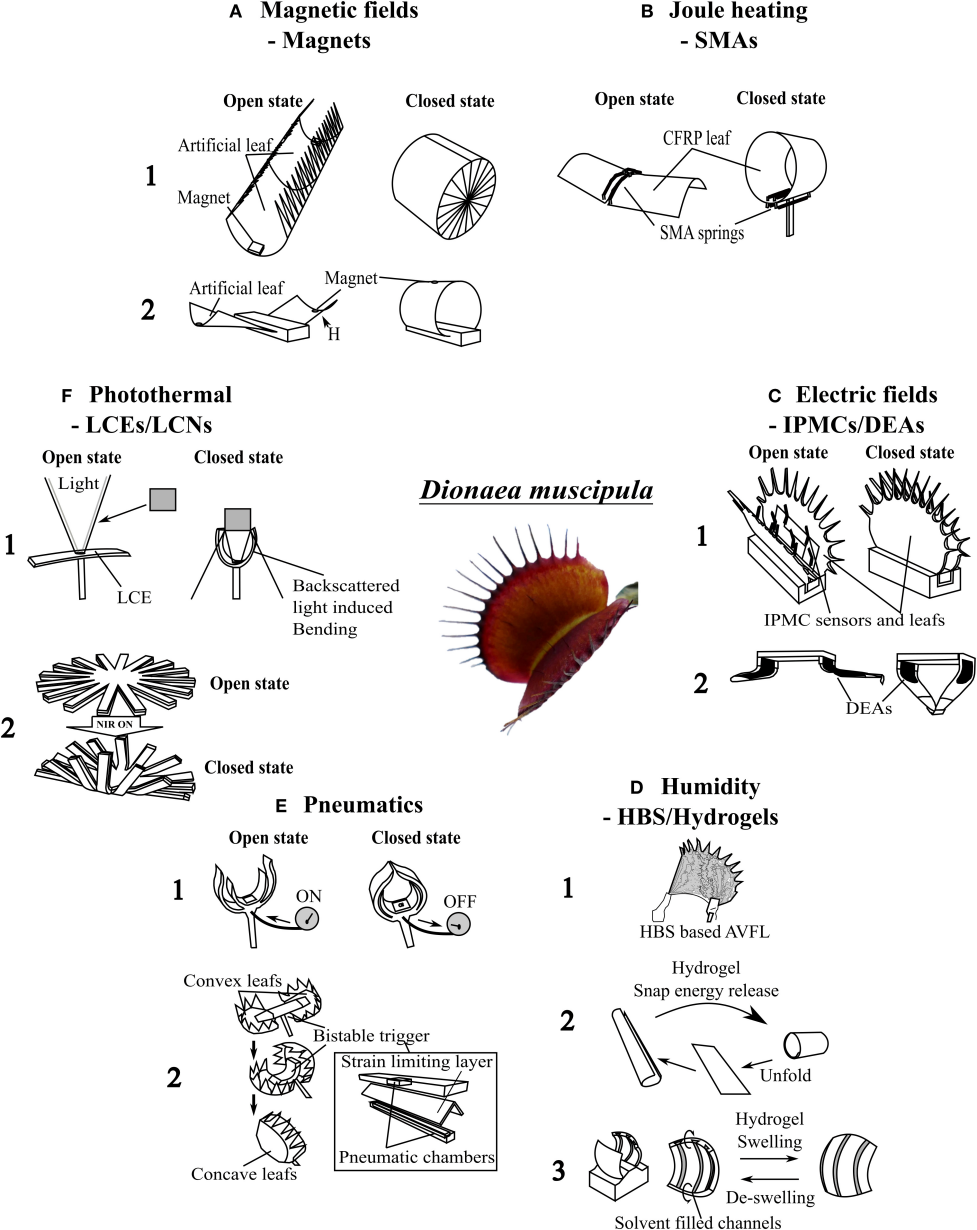
Overview of artificial Venus flytrap systems (AVFTs) categorized by actuation mode. Center: The biological role model *Dionaea muscipula*; its basic build and functionalities were abstracted into various AVFT systems. **(A)** Electromagnetic systems: (1) Electromagnetic CFRP-based AVFT (Zhang et al., 2016); (2) electromagnetic CFRP-based gripper (Zhang et al., 2019). **(B)** Heat-driven SMA-based AVFT (Kim et al., 2014). **(C)** IPMC-based systems: (1) IPMC-based AVFT with artificial trigger hairs (Shahinpoor, 2011); (2) DEA-based AVFT with a fast gripping motion (Wang et al., 2019). **(D)** Humidity-driven systems: (1) HBS-based humidity change-driven AVFT (Lunni et al., 2020); (2) hydrogel-based water- and temperature-triggered AVFT (Fan et al., 2019); (3) Hydrogel-based solvent-triggered doubly curved system [adapted from Lee et al. (2010)]. **(E)** Pneumatic systems: (1) 3D-printed pneumatic AVFT (Temirel et al., 2016); (2) silicone-based AVFT (Pal et al., 2019). **(F)** Photothermally driven systems: (1) LCE-based AVFT (Wani et al., 2017); (2) NIR-light-triggered AVFT (Lim et al., 2017). Sketches of the AVFTs are all originals based on the concepts presented in the mentioned references.

The unique functions of the Venus flytrap are of significant interest for biomimetic robotics, as indicated by the development of various artificial Venus flytraps (AVFTs) over the last 25 years (Figure 2). One of the first macroscopic systems was driven by DC motors (Venus flytrap robot) and developed by Yang et al. (2012) who transferred the theoretical models of prey capture into a first fully functional technological demonstrator for a detailed description of the biological role model, the reader is referred to papers by Forterre et al. (2005); Markin et al. (2008); Volkov et al. (2008, 2014); Yang et al. (2010, 2012), Poppinga et al. (2018), and Sachse et al. (under revision). The models theoretically describe the trap closure after prey detection by *D. muscipula*. Most other AVFT systems were soft robots based on smart materials systems, spanning from LCE-based systems of a few millimeters in size (Kohlmeyer and Chen, 2013; Wani et al., 2017) (Figure 3F) to more macroscopic designs driven by heat produced photothermally (Figure 2F) (Lim et al., 2017) or via joule heating (Figure 3B) (Kim et al., 2014; Lim et al., 2017). Other systems were actuated by magnetism and electricity (Figure 3A) (Shahinpoor and Thompson, 1995; Shahinpoor, 2011; Schmied et al., 2017) or pressurized air (Temirel et al., 2016; Pal et al., 2019) (Figure 3E) or were based on hydrogels activated via enzymes (Athas et al., 2016) and moisture (Lee et al., 2010; Fan et al., 2019) (Figure 3D). In addition, the aforementioned designs for applications in architecture can be scaled up to span widths of several meters such as in the Flectofold actuators for facade shading inspired by the trapping movement of the waterwheel plant *A. vesiculosa* (Körner et al., 2018; Saffarian et al., 2019). Furthermore, snap buckling, as seen in and inspired by the Venus flytrap, has been used in various applications from snap-through transitions in optical devices (Holmes and Crosby, 2007) to bistable buckling beam actuators for mechanical memories, micro-relays, micro-valves, optical switches, or digital micro-mirrors in, for example, MEMS systems (Saif and Taher, 2000; Park and Hah, 2008; Shankar et al., 2013).

The AVFT systems differ in their basic composition markedly from one another. In order to achieve better comparability, AVFT systems, representing the current state of the art, are categorized in terms of their actuation mode (Figure 3). In the following, we provide an overview of existing AVFT systems, highlight their advantages/disadvantages, and compare their performances, by using values from literature (providing that data are available) (Table 1). All AVFT systems should meet certain general conditions and requirements to be classified as an AVFT; these include actuation after a certain trigger, a certain closure time, snap buckling movement of the lobes, and reversibility. Influencing factors for these systems are costs for production and operation, weight, size, geometry, feasible temperature range, trigger parameters, energy consumptions, produced forces, and robustness. The following parameters are used for a comparison of AVFT systems not only among themselves but also with the biological role model: actuation type, sensing capabilities, usage of the snap buckling principle, lobe closure time, input/requirements for actuation, and reversibility of closure (Table 1). The comparison draws attention to current shortcomings and possible novel application fields of AVFT systems.

**Table 1:** Comparison of AVFTs with the biological role model with respect to various parameters.

**Schematic**	**Type**	**Actuation**	**Sensing**	**Snap buckling**	**Closing time**	**Input/requirements for actuation**	**Reversibility**
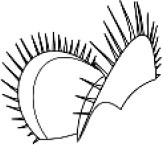	*Dionaea muscipula*	Stimulation of trigger hairs results in active water displacement	Touch-sensitive trigger hairs	Yes	0.1–0.5 s [1]	~300 μmol ATP (at standard conditions equals 9.66 J) [2]	Yes
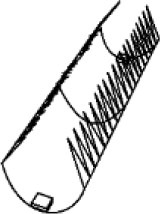	Magnet	Electromagnet	No sensor/actuated manually	Yes (no spatial inversion of configuration)	0.1 s [3]	Repulsive force by the electromagnet 0.06–41.46 N [3,4]	No/manually
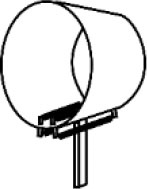	SMA	Electric current/ joule heating	No sensor/actuated manually	Yes (no spatial inversion of configuration)	0.1 s [5]	Closing: 12.4 J for 4.5 s reopening: 48 J for 10 s [5]	Yes
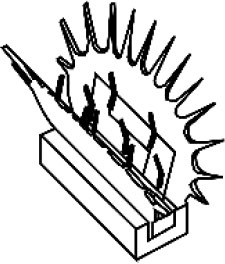	IPMC	Electric field/ voltage	Touch-sensitive IPMC-based trigger hairs [6]/proximity sensors [7]	No	0.05 s [7]	4–9 V of input voltage [6,7]	Yes
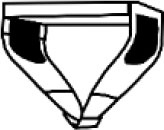	DEA	Voltage	No sensor /actuated manually	Yes	0.17 s [8]	6 kV, 7.7 mA for 0.04 s	Yes
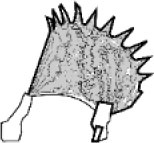	Hydroscopic bistable sheet	Swelling of hydroscopic layer	Inherent to the material	Yes	0.5 s [9]	Rise of relative humidity of 30%	Yes
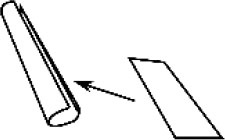	Hydrogel	Water with various temperatures	Inherent to the material	Yes (no spatial inversion of configuration)	30 s needed from contact with stimulus, <1 s for snapping [10]	Water with a temperature difference of 40 K [10]	Yes
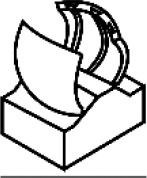	Hydrogel	Solvent	Inherent to the material	Yes	3.6 s needed from contact with stimulus, 0.012 s for snapping [11]	Solvent [11]	Yes
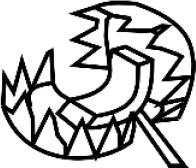	Pneumatic	Pressurized air	No sensor /actuated manually	Yes	0.05 s [12]	0.35–0.7 bar [12]	Yes
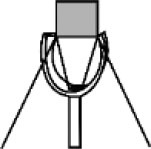	Photothermal—LCE	Light with certain wavelength	Inherent to the material	No	0.2 s [13]	Light with wavelength of 488 nm and intensity of 0.3 W [13]	Yes
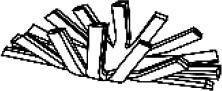	Photothermal—PEDOT/PDMS	Near-infrared light	Inherent to the material	No	~4 s [14]	Light with wavelength of 80 nm and intensity of 910 mW cm^−^^2^ [14]	Yes

*Sketches of the AVFTs are all originals based on the concepts presented in mentioned references*.

*References: [1] Forterre et al. (2005), [2] Jaffe (1973), [3] Zhang et al. (2019), [4] Zhang et al. (2016) [5] Kim et al. (2014), [6] Shahinpoor (2011), [7] Shi et al. (2012), [8] Wang et al. (2019), [9] Lunni et al. (2020), [10] Fan et al. (2019), [11] Lee et al. (2010), [12] Pal et al. (2019), [13] Wani et al. (2017), [14] Lim et al. (2017)*.

## Characteristics of the AVFT Systems (Actuation, Design, and Functionality)

As a common basis for a comparison, we describe here the characteristics of the various AVFT systems. A focus on the actuation mode, material composition, and lobe closure (movement and time) of the various AVFT systems has also enabled us to compare the systems with the biological role model.

Like most soft robots, two pneumatically driven AVFT systems exist that are also triggered by pneumatic actuation. Temirel et al. (2016) have developed a pneumatically driven 3D-printed AVFT (Figure 3E, 1). This system incorporates a touch sensor that is connected to a pneumatic controller. In sensing an object by touch, the shutoff valve is triggered, and the AVFT closes within 8 s; it reopens when pressure is applied. An increase in “trap lobe” displacement correlates with an increase of applied pressure (Temirel et al., 2016). Another pneumatic artificial trap presented by Pal et al. (2019) (Figure 3E, 2) is based on prestressed soft actuators (PSAs) and performs closing movements in 0.05 s. This is achieved through the release of stored elastic energy in different segments of the trap, namely, the spatially curved lobes and the prestressed backbone. Like the biological role model, *D. muscipula*, the artificial lobes invert their curvature from concave to convex while closing. The backbone that connects the two lobes consists of three layers, a prestressed silicon layer (which stores elastic energy) with the activation air chamber, a folded strain-limiting layer in the middle, and another silicon layer at the bottom with a second pneumatic channel for reopening of the AVFT. When pressurizing the activation air chamber, the folded strain-limiting layer is flattened, resulting in a decrease in flexural rigidity of the segment until the PSA “snaps” and the AVFT closes. The snapping motion can be reversed when pressure is applied to the lower pneumatic chamber, reopening the trap, and refolding the crease in the strain-limiting layer (Pal et al., 2019). As in other artificial traps, the pneumatically actuated trap does not feature a separated sensor and has to be activated manually.

One of the greatest advantages of the current AVFT systems is the possible contactless actuation, although most systems need to be triggered, like the pneumatic systems, by human input, such as the magnetically actuated artificial flytraps that use carbon-fiber-reinforced prepreg (CFRP) cylindrical shells as “leaves” and that are manually actuated with an electromagnet (Figure 3A) (Zhang et al., 2016, 2019). After activation of the electromagnet, the repulsive force between the electromagnet and a permanent magnet, which is attached to the outer leaf rim, triggers a snapping motion. The leaves have a positive curvature in the x-axis and no curvature in the y-axis. After actuation, closure is achieved within 0.2 s, whereas the curvature changes to a positive curvature along the y-axis and zero curvature along the x-axis (Zhang et al., 2016). Moreover, SMA springs are used to actuate a similar AVFT system based on CFRP lobes (Figure 3B) (Kim et al., 2014). When actuated by electric current, heat is generated within the material via joule heating, which causes the spring to change its structural phase from martensite to austenite and to shorten (Kumar and Lagoudas, 2008). Hereby, enough force is generated to overcome the crest of the potential energy hill of the system and results in a snapping movement that closes the artificial trap in 0.1 s. By using a second SMA spring as an antagonist, the process can be reversed (Kim et al., 2014). Both systems use external actuators attached to the lobes to drive the closure.

In using smart materials as a base material, AVFT system lobes have been developed that directly react to a stimulus with movement; in the case of the following examples of electroactive polymers as actuators, the stimulus is electrically based. One smart material type used to create artificial traps is a substance composed of ionic electroactive polymer metal composites (IPMCs) (Figure 3C) (Shahinpoor, 2011; Shi et al., 2012). The multilayer material performs bending movements when exposed to an electric field. Whereas, positive charges can move inside the polymer, negative charges are located at an immobile backbone that impedes their ability to move, causing a separation of charges in the electric field (Shahinpoor, 2011). Dissolved cations move within the material, dragging solvent along and causing one side of the material to swell and the opposite side to shrink. Furthermore, IPMCs can generate a small output current. When the material is bent by external force, the solvent is displaced, and the resulting charge separation generates the current. Based on this principle, the IPMCs can be used as bending sensors. Artificial traps made of IPMC attain closing times of around 0.5 s and feature a separated sensor made of the same material that connects input signals via an amplifying circuit with the actuator (Shahinpoor, 2011). These sensors are used to trigger the IPMC bending motion. Similar to IPMCs, dielectric elastomer actuators (DEAs), another type of electroactive polymers, react to an applied voltage. DEAs convert electrical energy into mechanical work. A DEA is a compliant capacitor in which a passive elastomer film is sandwiched between two compliant electrodes. When a voltage difference is applied between the electrodes, the opposite electrodes attract each other because of electrostatic forces (Maxwell stress) (Pelrine et al., 2000). The elastomer film is compressed in a vertical direction and expands in a lateral direction; this expansion actuates a bistable system. Wang et al. (2019) have applied this principle to actuate an AVFT gripper based on parabolic PET foil framing. On each side of the frame, a DEA is attached with a center electrode within the frame, connecting both DEAs. The system can be switched from one stable minimal energy state to another by manually applying a short high-voltage impulse (*V* = 6 kV, *I* = 7.7 mA for 0.04 s) (Wang et al., 2019). The DEAs attached to the bistable frame snap within 0.17 s, closing and opening the AVFT gripper. The total energy consumption for each grasping movement amounts here to ~0.14 J (with 0.003 s of charging time of the actuator; Wang et al., 2019). After snapping, no energy is needed to hold the position. The system has no sensing capabilities and requires manual triggering.

Human input is not the only means that can be used to trigger AVFT systems. Changes of environmental conditions such as humidity or temperature have been employed as input, for example, for hydrogel-based artificial traps (Figure 3D) (Fan et al., 2019; Lunni et al., 2020). These systems are based on composite (Fan et al., 2019) or hybrid hydrogels (Athas et al., 2016), utilizing various swelling behaviors and coefficients of the building components under variable environmental and triggering conditions for movement. Within hydrogels, sensing (e.g., sensing and reacting to changes in humidity) and acting (bending, folding, and snapping movement caused by swelling) are combined in one structural system. Athas et al. (2016) constructed, with a hybrid hydrogel, a rudimentary analog of the Venus flytrap, consisting of two flat gels as “leaves” connected via a folding hydrogel as a hinge or “midrib.” When exposed to a certain quantity of enzyme (50 U/ml collagenase), the hinge bends, and the leaves close within 50 min. The system of Fan et al. (2019) is faster in comparison and can perform a rapid snapping motion (<1 s) along the transversal axis. However, first, it has to be initialized by heating it in a water bath from 20 to 60°C and then keeping it at 60°C for 10 min; only after this treatment is the system ready to perform a fast snapping motion (Figure 3D, 2). The actuator is based on a reduced graphene oxide/PDMAEMA composite. By polymerizing the monomers with UV light from only one side, the light-exposed side features higher chain density and cross-linking density than the other side. When the actuator is submerged in water at 20°C and when the water temperature is raised to 60°C, the flat composite sheet bends toward the high-density side along the longitudinal axis; because of the shrinking of the high-density side, the system accumulates potential energy as stresses within the material. When placed back into water at 20°C, it takes 30 s to reverse the rollup motion slightly, followed by a fast snapping motion along the transversal axis (<1) (Fan et al., 2019). After snapping, the reopening through gradually unrolling back to a flat state takes 60 min in water at 20°C. In contrast to these two rather slow systems, Lee et al. (2010) have developed a 3D polymeric device that snaps open in response to a solvent within 3.6 s and is able to snap close again. The system consists of two 3D-printed, doubly curved hydrogel sheets connected via a flat hydrogel sheet forming a table-like structure (Figure 3D). Lee et al. used poly(ethylene glycol) diacrylate (PEGDA) as a base material to produce this π-shaped structure via 3D hydrogel printing. On the inside of the convex sheets lie three parallel-aligned channels with a trapezoidal cross section for solvent transport. When solvent comes into contact with a sheet, it is transported within the microfluidic channel network by capillary action over the entire length of the sheet. Local swelling around the aligned channels causes the doubly curved device to bend only along the vertical axis (Lee et al., 2010). Thereby, the elastic sheet is only stretched along one axis storing elastic energy (attributable to the bending–stretching coupling of the doubly curved plate geometry, Lee et al., 2010). Through further swelling, the sheet deforms and passes through the energy barrier. Stored elastic energy is instantaneously released and converted into kinetic energy; as a result, an outwardly directed snap buckling opening occurs (Lee et al., 2010). During drying and de-swelling, the system reverses its movement and snaps back into its original shape. The whole opening and closing process takes places within 5 s. The snapping motion of the sheets in the de-swelling phase takes 12 ms. The system releases 25.5 nJ of energy during the snapping motion and, thus, is able to propel itself 7 mm into the air (Lee et al., 2010). This system is able to snap open and close in response to solvent as stimuli. A purely humidity-responsive AVFT leaf based on a hygroscopic bistable sheet (HBS) system has been developed by Lunni et al. (2020) (Figure 3D). The system consists of a pre-stretched passive layer of polydimethylsiloxane (PDMS) and a hygroscopic active layer of electrospun polyethylene oxide (PEO) nanofibers. The PEO swells in response to a rise of environmental humidity of around 30%. The coupling between the hygroscopic material and the passive layer causes a curvature reduction of the system until it snaps within 0.5 s (Lunni et al., 2020). The initial state can be restored by reducing the humidity.

In contrast to the above systems, light-driven monolithic LCE-based artificial traps have sensing and actuating mechanisms combined in one material (Figure 3F). When an LCE-based AVFT is exposed to light of a certain wavelength, a cis-trans-isomerization of photoactive molecules within the LCE leads to a change in length of the top layer and therefore to a bending motion and a closure within 0.2 s. After the light source is removed, the actuator returns to its original shape (Wani et al., 2017). The LCE actuators are also temperature responsive. If the energy provided by a heat source is sufficient to trigger the isomerization, bending occurs even without light. This heat-driven motion is also reversible. Wani et al. (2018) presented a second demonstrator that uses liquid crystal networks (LCNs) as photo actuators whose reaction can be controlled and modified by light and humidity. By using these humidity-controlled photo-actuators, an artificial nocturnal flower was developed that closed during the day (conditions: low humidity levels and high light levels) and opened at night (conditions: no light and high humidity levels). The LCN humidity-gated photo-actuators could be actuated with lower light intensities than their photothermal LCE actuator counterparts (Wani et al., 2017, 2018). Another photothermal AVFT, developed by Lim et al. (2017), is actuated via near-infrared (NIR) light at a wavelength of 808 nm (Figure 3F, 2). This bimorph structure consists of a photothermal PEDOT layer and a soft PDMS layer. A heat pocket inside the structure is created by exploiting the photothermal properties of PEDOT. When actuated by NIR light, bending occurs, and the trap closes in <4 s and reopens when the infrared light source is removed. This system shows reversibility but not the typical snapping motion of the Venus flytrap.

## Comparison of AVFT

To evaluate such systems in direct comparison to *D. muscipula* and to determine whether they are truly AVFTs, one can imagine using a Turing test (Pinar Saygin et al., 2000). Turing's aim was to provide a method to assess whether or not a machine can “play the imitation game.” A tester has to determine if either a human or a computer program has given him an answer to a question. If the tester is not able to distinguish the human from the machine, then the machine or program can be viewed as having an artificial intelligence (Pinar Saygin et al., 2000). Within such a test involving the AVFT, distinguishing criteria would include the basic functionalities, appearance, and behavior of the artificial vs. the biological model. The biological role is able to harvest and store energy from the environment, to sense and compute sensed information, and to react accordingly. For example, the plant can sense prey and close its lobes in reaction to triggers but can also sense damage and repair or discard the damaged part. The general Bauplan of the two lobes with sensors and harvesting structures have to be fulfilled. If all these criteria are met, one should not be able to distinguish the artificial from the real Venus flytrap in its reactions and mode of functioning. The target performance of the truly artificial system would be defined as being able to sense “prey,” respond to it with flap closure, adapt to a changing environment, and harvest and store energy, if the general Bauplan and appearance of the biological role model is maintained.

The comparison provided within this review is a first baseline for such a Turing test concerning functionalities. As none of the systems is currently able to harvest and store energy from the environment, we focus here on the key features characterizing the closing motion of the biological Venus flytrap and AVFT systems. These are highlighted in Table 1, enabling a more direct comparison of the systems and directly showing whether the state-of-the-art systems are capable of meeting the requirements of an AVFT: actuation after a certain trigger and with a certain closure time, type of movement (snap buckling), and reversibility. Of note here is that a comparison involving the input or required energy for actuation is only possible and feasible in specific cases because of the variable energy forms and inputs used for the actuation.

The magnetically driven and SMA systems are based on the same basic principal and material, namely, CFRP cylindrical shells as “leaves” that perform a curvature change within 0.1 s when actuated manually. The closing speed is within the range of the biological role model. These systems do not have sensory capabilities, nor is the curvature change a spatial inversion as seen in *D. muscipula*. The initial configuration can be restored in the SMA-based system by an antagonist function. Low force and energy are required to initiate the snap buckling within the systems. The SMA requires slightly more energy for closure than *D. muscipula* (12.4 J, (Kim et al., 2014), vs. 9.66 J in the natural system, Jaffe, 1973).

Being able to be triggered by changing environmental conditions, the hydrogel-based, HBS-based, and photothermal systems have a sensing capability inherent to their composition. Their base material reacts to humidity/moisture (HBS and hydrogel), light (LCEs), and temperature changes with a conformational change within the material. The LCN nocturnal flower (Wani et al., 2018) utilizes all triggering conditions (humidity, light, and temperature) but is also far removed from the biological role model as no curvature change, snap buckling, or fast actuation (1.8–9 s for closure) occurs. In contrast, the hydrogel-based system developed by Fan et al. (2019) is able to perform a fast snapping motion but shows a long initialization phase to snapping (30 s from stimulation with a temperature change of 40 K). Moreover, the jumping hydrogel of Lee et al. (2010) has an initialization phase, in which elastic energy is built up over 3.6 s through controlled swelling, until the system snaps open within 12 ms. Via de-swelling, the system snaps close again, releasing stored energy, and propelling the system into the air. The HBS-based system of Lunni et al. (2020) represents a system that is able to perform a fast (0.5 s) and reversible snapping motion in correspondence to a humidity change of 30%. This system performs the fastest moisture-driven motion without an initialization phase of all of the AVFTs and resembles the biological role model not only in appearance but also in motion. The LCE-based AVFT by Wani et al. (2017) can be triggered via a change in environmental conditions, but the system resembles a Venus flytrap only in a purely reactive way by being able to “sense” its “prey.” If “prey” enters the space between the lobes, it reflects the emitted light of the central rod, illuminating the LCE lobes, which then bend and catch the “prey” within 0.2 s. This system can also be utilized as a gripper, automatically gripping an object whenever it lies between the lobes. The energy required to activate the systems is again far removed from the biological role model (temperature change of 40 K and light of an NIR laser at 0.3–1.1 W with an intensity of 980 mW cm^−2^). The PEDOT/PDMS bimorph-based AVFT of Lim et al. (2017) cannot be considered an AVFT in the proper sense, as the system incorporates none of the basic principles or predefined requirements. However, the system highlights the possibility of usage underwater and as an oscillator or light-driven motor.

The pneumatic system developed by Pal et al. (2019) is able to change the curvature of its lobes, perform a snap buckling motion, and close within 0.05 s, making it faster than the biological role model. The system has no sensors, but by using antagonistic pneumatic chambers, the motion can be reversed. The low-energy fast AVFT gripper system developed by Wang et al. (2019) is based on DEAs. Via the combination of a bistable parabolic-shaped PET foil backbone, the DEA can switch within 0.17 s from one stable state to another actuated by a short electrical impulse resulting in low-energy consumption (~0.14 J). Like the biological role model, the system does not require energy to be held during an open or closed state. On the basis of the transfer of movement principles, these two systems represent the most sophisticated AVFT systems developed so far.

The only system incorporating a sensing system similar to that of the biological role model is the IPMC-based AVFT of Shahinpoor (2011). The IPMC trigger hairs are attached to the IPMC lobes and connected to a solid-state relay. When the trigger hairs are deflected by an object, an electrical signal is generated, which is used to activate a small dynamic voltage generator actuating the IPMC lobes (Shahinpoor, 2011). However, neither curvature change nor snap buckling is performed within the closure movement.

None of the above-described system transfers all principles of *D. muscipula* into an artificial system. Of note here however is that the aim of most of these studies was not to transfer the principles fully into one system but to highlight a novel actuator, material, or bistable system and to build with it a system that resembles a Venus flytrap. To transfer all essential principles behind a *D. muscipula*, one needs to develop a system that is able not only to snap and move like the role model but also to sense its environment and “prey,” to make a decentralized decision to capture “prey,” and to harvest energy both via prey capture and from the environment.

## Envisioning a True AVFT as an Inspiration for Living Adaptive Materials Systems and Novel Technologies

The presented AVFT systems highlight the great potential that lies within bioinspired and especially plant-inspired soft machines in the field of adaptive and autonomous systems. Some of the systems are able to sense changes in the environmental conditions or approaching “prey” and react to them via actuation. These “trapping” reactions are achieved via electricity, thermally, pneumatically, or magnetically, or by humidity-change-driven actuators. In this way, systems are constructed that can harvest energy from the environment for the actuation in the case of the humidity-driven and photothermally driven systems.

**Figure 4:**
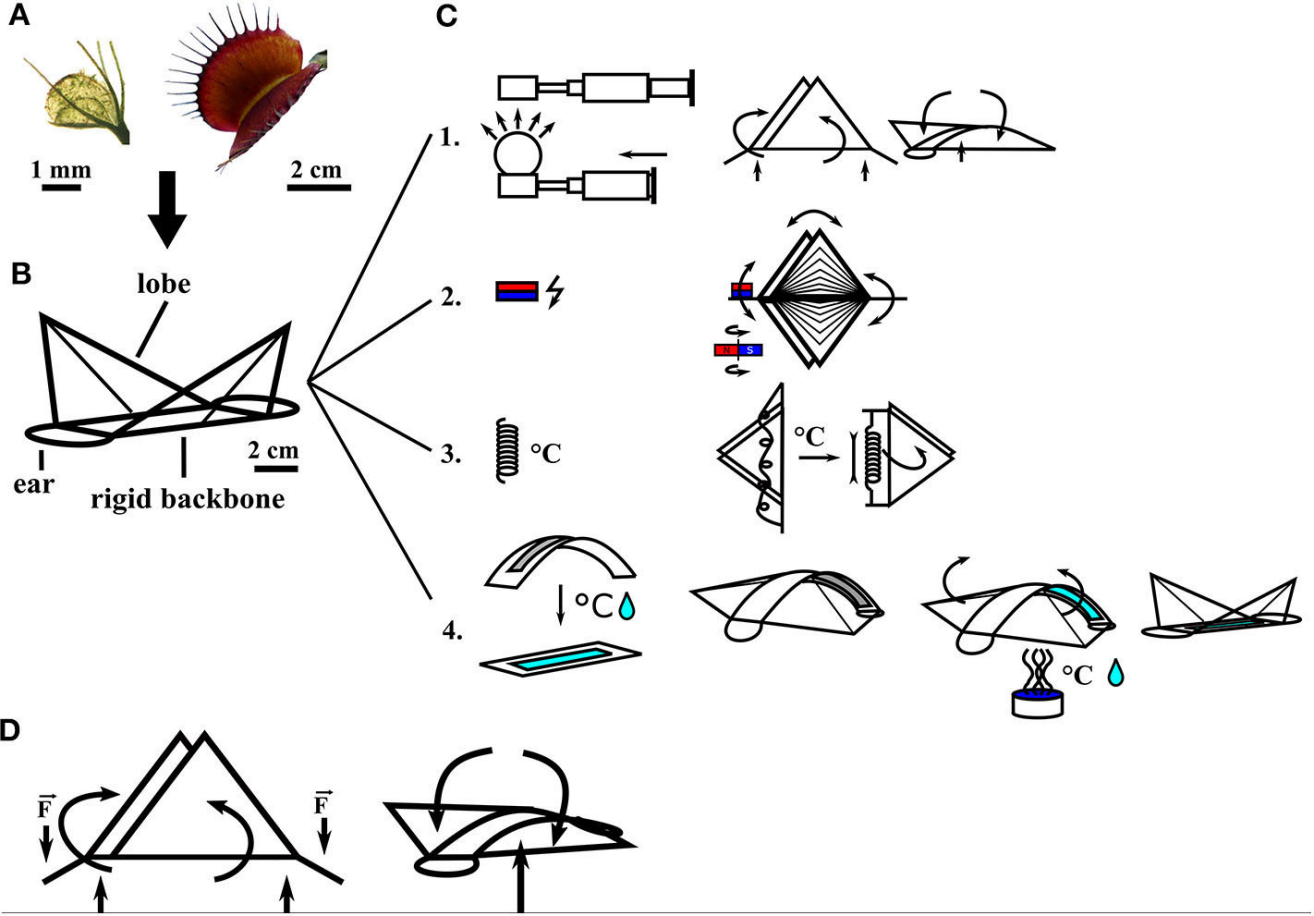
Compliant foil AVFTs with four different actuation modes. Biological role models *Aldrovanda vesiculosa* (left) and *Dionaea muscipula* (right) **(A)** are abstracted into a compliant foil system **(B)** with two lobes, two ears for actuation, and a rigid backbone. Various movement actuators **(C)**: (1) Pressurized pneumatic cushion (left) pushes the backbone upwards, closing the lobes (middle); when pressure is applied via a central cushion, the backbone bends, and the AVFTs snap open (right). (2) Magnetic field actuation of closure movement; a permanent magnet is attached to one ear and actuated via a rotating magnetic field bending the ear up and down, closing and opening the AVFT, respectively. (3) An SMA spring is attached to the ears behind the backbone; when the SMA is heated in a contact-free manner via a rise in environmental temperature, the spring contracts, closing the AVFT. (4) Via specifically designed 3D-printed backbones coated with hydrogel (left), the system can be held in the snap-opened state, until the system is initialized via a stimulus combination of humidity and temperature (right). **(D)** Movement principle of the AVFT system: bending down the ears closes the lobes, and the system snaps open by bending the backbone.

The above-described material-wise, often sophisticated, systems inspired our low-cost, low-energy, fast-moving, simplified AVFT system (Esser et al., 2019). This system highlights the status quo of AVFT actuation within one system and is currently being characterized. The basic geometry of the snap traps of the Venus flytrap (*D. muscipula*) and waterwheel plant (*A. vesiculosa*) (Poppinga et al., 2018; Westermeier et al., 2018, 2019; Sachse et al., under revision) was abstracted in a compliant foil demonstrator with two triangular lobes connected via a rigid backbone with two ears for actuation (Figure 4B). By applying a force to the ears and bending them down, the geometrically connected lobes are made to close (Figure 4D). The movement can be actuated pneumatically (Figure 4C, 1), thermally (through SMAs) (Figure 4C, 2), or magnetically (Figure 4C, 3), and a hydrogel-based locking mechanism can be incorporated into the system (Figure 4C, 4). The system can snap shut (Venus flytrap) or continuously bend to close (waterwheel plant) like its biological role models and is able to snap open in a snap buckling motion of its backbone. Through the hydrogel and specifically designed 3D-printed backbones, the system can be held in the snap-opened state, until the system is initialized via a stimulus combination of humidity and temperature.

This snap trap demonstrator is considered to be a baseline for the development of a true AVFT. The final system will be able to react to certain triggers, adapt to the environment, and harvest energy to maintain its homeostasis, implying that the energy demand of the actuation systems is lower or equal to the provided energy from the harvester and storage structures. In order to attain this, materials are needed that are able to sense, react, and adapt to the environment (Walther, 2019). These should be able not only to adapt but also to learn and to transition from one stable energy state to another. They must be able to cope with local triggers and convert them into global answers or adaptations. As in nature, the materials and systems need self-healing properties and damage-sensing and damage-control capabilities. These might be achieved via chemically or catalyst-based and diffusion-based information transfer, as in the stimulus and immune responses of plants (Spoel and Dong, 2012). Additionally, a decentralized decision-making process should be incorporated that decides the time to act and the specific stimulus for action. To achieve autonomy in these systems, energy must be harvested from the environment and stored and distributed within the system. The system should also be sustainable and easily recycled to conform to the agenda 2030 of sustainable development (Colglazier, 2015).

In our opinion, a multilayer materials-based system is best suited to cope with all these requirements and specifications. The outer layers should be compliant self-healing foils, safeguarding inner systems from harsh environments and repairing any damage occurring during use. These layers should contain an intermediary layer of stimulus-computing metamaterials with embedded energy-harvesting and storage materials systems. Of note here, stresses, strains, and deformations must be deflected or guided around the energy-generating regions within this layer. The central actuation layer should consist either of an environmentally triggerable active material connected to the environment directly or of sensors lying on the outer layers and gaining the energy for actuation from the harvesters. These systems would be able to adapt to changing weather conditions by altering the lobe curvature for better light incidence; to adapt to variable “prey” dimension by altering their inner structure to achieve stiffening, elongation, or higher flexibility; and to heal damage caused by prey or harsh conditions. The technology to build the components for such a compliant multilayer system is partially available today. Currently, self-healing foils (Hönes et al., 2017), flexible sensors, and electronic circuits (Lu and Kim, 2014; Majidi, 2018; Kumar et al., 2019), solar batteries (Zhong et al., 2017), and, as shown above, smart actuators can be produced using, for example, multiphoton lithography (Malinauskas et al., 2009; Vaezi et al., 2013; Meza et al., 2014, 2015), wafer technology (Kim et al., 2012; Segev-Bar and Haick, 2013), spray coating (Kent et al., 2020), and 3D and 4D printing (Kumar et al., 2019; Ma et al., 2019). Nevertheless, one challenge that remains open is the combination of all these components into one multi-materials system. Therefore, the development of a fully functional AVFT with the aforementioned specifications will involve the development of novel materials systems. These systems will enable the manufacture of a new phase of self-sensing and environment-adaptive robots. Such advancements will lead to innovative technologies, as unprecedented types of materials systems will have to be developed for their production.

## Future Application of AVFT as a Novel Gripping Technology

In robotics, an artificial flytrap can serve as a deployable structure, such as gripper or energy harvester, which can be attached to a fixed structure to perform independent functions and to increase overall system flexibility and adaptation (Yang et al., 2012). A first glimpse of these possible usages is given by the CFRP- and DEMES-based AVFT gripper systems from Wang et al. (2019) and Zhang et al. (2019) consisting of DEMES-based and CFRP-based smart materials systems, respectively. However, one shortcoming of these systems is that the materials cannot adapt to the gripped object. Because of their flexibility, they do not destroy the payload but unfortunately also do not adapt to it to achieve a better grip. In order to use AVFT as low-energy grippers, robust materials with adjustable stiffness are required that are able to actuate the system consistently and to adapt, on demand, their stiffness to the requirements of the payload. A material combination that might be able to meet these requirements is a combination of soft elastomers with fluidic channels filled with liquid metals or low-melting-point alloys (LMPAs), as are used for soft sensors (Yufei et al., 2017). Sensor hairs consisting of triboelectric materials or IPMCs (Shahinpoor, 2011) might be used to identify payload properties and trigger an adaption process within the material of the gripper lobe. LMPA integrated into the lobe material might stiffen and thus strengthen the materials system via on-demand temperature changes, liquefaction, or curing. A combination of adaptive stiffening materials with flexible miniaturized energy harvesters such as photo-batteries or material immanent triboelectric and thermoelectric harvesters should enable the next generation of grippers to act as autonomous systems.

For plant-inspired robotics, these systems could be employed as attachment, manipulation, or energy-harvesting structures within harsh environments. In order to enable such systems to cope with harsh environmental conditions, these systems must run with low wear and low to no maintenance requirements because of, for example, their low complexity. To achieve this, the proposed systems should be able to repair damage and heal themselves, as their natural role models do (Speck and Speck, 2019). The incorporation of a dissolvable sacrificial layer (SL) underneath the outer layer of the multilayer would be a possible solution (Hönes et al., 2017). If the outer layer is damaged, the SL would be exposed and dissolved by moisture or atmospheric gases (oxidation), which would remove the support for the damaged layer, detaching it and renewing the functional surface of the outer layer.

A combination of the aforementioned principles and functionalities will lead to autonomous low-energy systems with embodied energy and intelligence or with morphological computation (Paul, 2006; Polygerinos et al., 2017). As in nature, these systems will achieve tasks not via the high computational power of today's robots but via their material composition, which will enable the system to start/stop moving or to grasp by design rather than by following a computer program. This achievement will reduce system complexity and maintenance requirements. A few examples of soft grippers able to adapt to the payload are indeed available and are capable of, for example, grasping a flower or an egg because of stiffness differences (Ilievski et al., 2011; Krahn et al., 2017; Wang et al., 2018). This principle has been inspired by the natural design of combined sensor and actuator systems (e.g., muscles) in animals (Paul, 2006; Polygerinos et al., 2017). For the design of materials systems that embody intelligence and are able to learn and transfer information, unit-cell-based mesostructured, and metamaterials systems with simple logical structuring might be employed (Grigorovitch and Gal, 2015; Meza et al., 2015; Haghpanah et al., 2016; Raney et al., 2016; Paoletti et al., 2017; Guseinov et al., 2020; Jin et al., 2020). Research into and the development of biomimetic artificial systems, such as AVFT systems, should lead to the creation of lifelike, adaptive, autonomous materials systems. In turn, these materials will spawn novel technologies such as autonomous grippers and resilient, adaptive, and low-maintenance solar harvesters for plant-inspired robots and self-charging sensors, smart phones, or electric vehicles, plus energy harvesters and adaptive shading for low-energy buildings and sustainable architecture.

## Author Contributions

TS and FE initiated and supervised the study. FE and PA performed the data collection from literature and wrote the first draft of the manuscript. All authors designed, performed, evaluated the study, critically revised, and approved the final version of the manuscript.

## Conflict of Interest

The authors declare that the research was conducted in the absence of any commercial or financial relationships that could be construed as a potential conflict of interest.
